# Inhibition of the JAK/STAT Signaling Pathway Suggests a Protective Effect against Acantholysis in Pemphigus

**DOI:** 10.1007/s10753-025-02417-y

**Published:** 2026-01-05

**Authors:** Farzan Solimani, Julia Holstein, Katharina Meier, Luz Maria Cano Rochin, Alberto Mesas-Fernandez, Yun-Fei Jiang, Maria A. Feoktistova, Morna F. Schmidt, Amir S. Yazdi, Franz Joachim Hilke, Kamran Ghoreschi

**Affiliations:** 1 Department of Dermatology, Venereology and Allergology, Charité - Universitätsmedizin Berlin, corporate member of Freie Universität Berlin, Humboldt Universität zu Berlin, and Berlin Institute of Health, Berlin, Germany; 2 Berlin Institute of Health at Charité - Universitätsmedizin Berlin, BIH Biomedical Innovation Academy, BIH Charité Clinician Scientist Program, Berlin, Germany; 3 Department of Dermatology, University Medical Center Tübingen, Eberhard Karls University, Tübingen, Germany; 4 Department of Dermatology, RWTH Aachen University, Aachen, Germany

**Keywords:** Pemphigus, Janus kinase inhibitors, Autoimmune blistering diseases, Acantholysis

## Abstract

Pemphigus is a severe autoimmune blistering disorder of skin and mucosa, which is elicited by gG autoantibodies targeting desmosomal components such as desmoglein 3 (Dsg3). Keratinocytes lose cytoarchitectural stability and reciprocal adhesion in a process called acantholysis, which manifests as blisters and erosions. It is widely accepted that keratinocytes themselves can produce inflammatory factors such as cytokines in response to mechanical stress. This suggests that stress as induced by the binding of autoantibodies to Dsg3 might initiate similar effects in keratinocytes. In this work, we evaluate the cytokine response of human epidermal keratinocytes after treatment with anti-Dsg antibodies (AK23 and human pemphigus IgG). Quantitative gene expression (qPCR) was performed to study cytokine expression induced after AK23 treatment. Activation of the signal transducer and activation of transcription (STAT) factors and the impact of Janus kinase (JAK) inhibitors in anti-Dsg antibodies-treated keratinocytes were evaluated by Western blotting and gene expression assays. Moreover, we studied the functional role of JAK inhibitors during anti-Dsg antibody-induced cell dissociation by dispase assay. Epidermal activation of STATs in pemphigus and control skin was determined by immunohistochemistry. Finally, a pemphigus lesion in a steroid-unresponsive patient was treated with topical ruxolitinib, the clinical response and epidermal STAT activation were assessed. Our investigation revealed that loss of epidermal integrity by AK23 is accompanied by increased expression of cytokines (*IL6*, *IL19*, *IL24*, *IFNE*) and anti-Dsg antibodies activate STAT1 and STAT3. Administration of JAK inhibitors in vitro and in vivo prevented anti-Dsg antibody-induced STAT activation and cell dissociation in keratinocytes.

**Supplementary Information:**

The online version contains supplementary material available at 10.1007/s10753-025-02417-y.

## Background

Pemphigus is an autoimmune skin disorder that develops in individuals with circulating autoantibodies against the epidermal structure proteins desmoglein (Dsg) 1 and 3. Dsg1 and Dsg3 are crucial components of desmosomes and provide cytoarchitectural stability of keratinocytes [1]. Following autoantibody binding to Dsg, keratinocytes lose cell-cell contact integrity in a process called acantholysis, which clinically manifests as blisters and erosions of the skin [2]. In pemphigus, autoantibodies alone can generate pathological skin changes without evident support of other cell types, as demonstrated in disease models [3]. The mechanism behind autoantibody-mediated acantholysis is not fully elucidated. It is likely that physical (i.e. mechanical stress) and cellular (i.e. inflammatory signals) factors are implicated. Other mechanisms than direct physical interference (steric hindrance) with Dsg like activation of intracellular signaling pathways seem to be involved in keratinocyte disruption [4]. Examples are the p38 mitogen-activated protein kinase (MAPK) [5, 6] protein kinase C (PKC) and Rho GTPases family proteins [7–9]. Beside their barrier function, keratinocytes show some immunological properties. Keratinocytes have a number of cytokine receptors and pattern recognition receptors (PRR) that they use to respond to external factors or inflammatory signals delivered from immune cells. Stimulated keratinocytes can produce a broad range of factors such as antimicrobial peptides and cytokines [10]. Keratinocytes have been suggested to produce Interleukin (IL) 1 family members, IL-20 family cytokines, type I-III interferons (IFN), transforming growth factor (TGF) family members and others such as IL-6, IL-7, IL-15, and tumor necrosis factor (TNF) [10]. Certain cytokines, such as the members of the IL-10 cytokine family, but also IL-6 and IFNs can act in an autocrine or paracrine manner and signal through activation of the Janus kinase (JAK) and signal transducer and activation of transcription (STAT) signaling pathway [11]. Increased STAT activation in keratinocytes has been observed in some common skin disorders. For example, STAT3 activation is typically found in keratinocytes during psoriatic inflammation. However, little is known about the activation of STAT proteins in keratinocytes during rare diseases like pemphigus [12]. Previously, we reported that *IL6*, *IL19* and *IL24* are overexpressed in lesional skin of pemphigus patients [13]. Since the aforementioned cytokines can be produced by keratinocytes, this suggests that the JAK/STAT pathway might contribute to pemphigus pathology. Here, we aimed to investigate the effects of anti-Dsg3 antibodies binding on cytokine expression and STAT activation in keratinocytes. We provide evidence that autoantibody binding leads to STAT activation and cell dissociation, which can be reversed by Janus kinase (JAK) inhibitors.

## Methods

### Samples

Diagnosis of pemphigus of patients whose samples (sera and/or skin) were included in this study was based on clinical presentation, positive direct immunofluorescence from perilesional skin showing epidermal intercellular IgG deposition and detection of anti-Dsg antibodies by enzyme-linked immunosorbent assay (ELISA, Euroimmun, Lübeck, Germany). The demographics of the pemphigus patients participating in this study are shown in Table S1−3.

### Keratinocyte Culture

Normal human epidermal keratinocytes (NHEK) were isolated from discarded healthy foreskin tissue by separating the epidermal layer from the dermis. Therefore, tissue pieces were incubated for 24 h with dispase II (Roche, Mannheim, Germany) and the epidermis was separated. By incubating the epidermal layer with Trypsin/EDTA solution (Merck, Darmstadt, Germany) for 30 min at 37 °C, single cell suspension was achieved and cells were cultured in CnT-07 medium containing 10 mg/ml gentamycin and 0.25 mg/ml amphotericin B (CELLnTEC, Bern, Switzerland) at 37 °C in a humidified atmosphere with 5% CO2. Experiments were performed at passages 2 to 4 and NHEK were previously differentiated for 24 h in the presence of 1.8 mM CaCl2. The isolation of primary human cells was approved by the medical ethical committee of the Eberhard Karls University (protocol 331/2010BO2) and performed in accordance with the Declaration of Helsinki principles.

### Western Blot and Immunodetection

For time course experiments, NHEK were incubated with 20 µg/ml AK23 or 1 U/ml purified IgG from sera from patients with active pemphigus vulgaris (PV IgG) for the indicated time points. Inhibitors were pre-incubated for 1 h at a concentration of 1 µM or 4 µM followed by the addition of cytokines (50 ng/ml, all Bio-Techne, Minneapolis, USA) for 30 min, 20 µg/ml AK23 for 4 h or 1 U/ml PV IgG for 2 h. Cells were lysed in Triton X-100 lysis buffer containing protease and phosphatase inhibitors (Roche, Basel, Switzerland). Equal amounts of total protein were separated by SDS-PAGE and transferred to a PVDF membrane. For specific protein identification, the membrane was blocked for 1 h at room temperature in LI-COR blocking buffer and incubated over night at 4 °C with primary antibodies recognizing actin (clone C4, 1:5000, Merck, Darmstadt, Germany or clone 13E5, 1:1000, Cell Signaling, Danvers, USA), phospho-STAT1 (Tyr701, clone 58D6, 1:1000), phospho-STAT3 (Tyr705, polyclonal, 1:1000), phospho-STAT4 (Tyr693, clone D2E4, 1:1000), phospho-STAT5 (Tyr694, clone C71E5, 1:1000), phospho-STAT6 (Tyr641, polyclonal, 1:1000), phospho-p38 MAPK (Thr180/Tyr182, clone D3F9, 1:1000), STAT1 (polyclonal, 1:1000), STAT3 (clone 79D7, 1:2000), STAT5 (clone D2O6Y, 1:1000, all Cell Signaling, Danvers, USA) or Dsg3 (clone 5H10, 1:1000, Bio-Techne, Minneapolis, USA), followed by detection with Dye-labeled secondary antibodies (680RD and 800CW, 1:14000, LI-COR, Lincoln, USA) for 1 h at room temperature. Visualization of protein bands and semi-quantitative analysis of signal intensity was performed with the Odyssey Sa infrared imaging system and Image Studio Lite software (LI-COR, Lincoln, USA).

### Dispase-based Keratinocyte Dissociation Assay

NHEK were pre-incubated for 1 h with indicated inhibitors at indicated concentrations in DMSO or DMSO alone (control condition) prior to the addition of 20 µg/ml AK23 or buffer for 4 h. Human PV IgG or control IgG were incubated for 24 h at a concentration of 1 U/ml, where one U equals the amount of PV IgG required to achieve a similar fragmentation as 20 µg/ml AK23. Detaching of the monolayer and application of mechanical stress were performed following the protocol provided by Schmidt et al. [14]. Generated fragments were fixed with 4% formaldehyde, stained with crystal violet over night at 4 °C and the number of fragments was subsequently counted. Experiments were conducted with three technical replicates per condition.

### Reagents

A list of reagents used for the experiments can be found in Appendix S1.

### Next Generation Sequencing and Bioinformatics

Total RNA from skin samples was isolated and submitted to c.ATG (NGS competence center Tübingen, Tübingen, Germany) for next-generation sequencing analysis as previously described [13]. 

### Quantitative Real-time PCR (qPCR)

NHEK were treated with 20 µg/ml AK23 for 4 h or pre-incubated with JAK inhibitors for 1 h before addition of IL-17 A and IL-22 for 30 min (50 ng/ml, Bio-Techne, Minneapolis, USA). Total RNA was isolated using the peqGOLD Total RNA Kit (VWR, Radnor, USA) following the manufacturer’s instructions. Reverse transcription into cDNA was performed with the Maxima First Strand cDNA Synthesis Kit for RT-qPCR (Thermo Fisher Scientific, Waltham, USA), according to the manufacturer’s protocol. Relative gene expression was determined by qPCR using TaqMan probes (all designed by TIB MolBiol, Berlin, Germany) and Probes Master for the Roche LightCycler480 system (Roche, Mannheim, Germany). *ACTB* and *POLR2A* were used as reference genes. Relative mRNA expression was calculated according to the 2^−ΔΔCq^ method. Sequences for primers, probes and qPCR parameters can be found in the appendix S1.

### Immunohistochemical (IHC) Staining of Skin

4 mm punch biopsies from lesional skin of patients with psoriasis or pemphigus were collected. Healthy skin samples were obtained from control patients, who underwent surgical procedures for non-inflammatory diseases. Each patient signed an informed consent and ethical approval was obtained by the ethics committee of the Eberhard Karls University Tübingen (protocols 309/2017BO2 and 759/2016BO2) and of the Charité – Universitätsmedizin Berlin (protocol EA4/194/19). The formalin-fixed paraffin embedded skin samples were cut into 4 μm sections and IHC staining was performed using an automated immunostainer (Ventana Medical Systems, Tucson, USA) according to the manufacturer’s protocol. Specific antibodies for phospho-STAT1 (Tyr701, clone 58D6, 1:500) and phospho-STAT3 (Tyr705, clone D3A7, 1:400) were obtained from Cell Signaling, Danvers, USA. Appropriate positive and negative controls were used to confirm the adequacy of the staining. Semi-quantitative analysis of the stained skin was performed using the color deconvolution plugin of ImageJ Fiji software according to Crowe et al. [15, 16]

### Statistics

Data were analyzed and plotted using GraphPad Prism software (Version 10.50.0, San Diego, USA). Statistical analyses were performed by using Dunn’s multiple comparisons test after Friedman test, Wilcoxon matched-pairs signed rank test and Dunn’s multiple comparisons test after Kruskal-Wallis test. Values of *p* < 0.05 were considered significant.

## Results

### AK23 Binding To Primary Keratinocytes Induces Inflammatory Responses

**Fig. 1:**
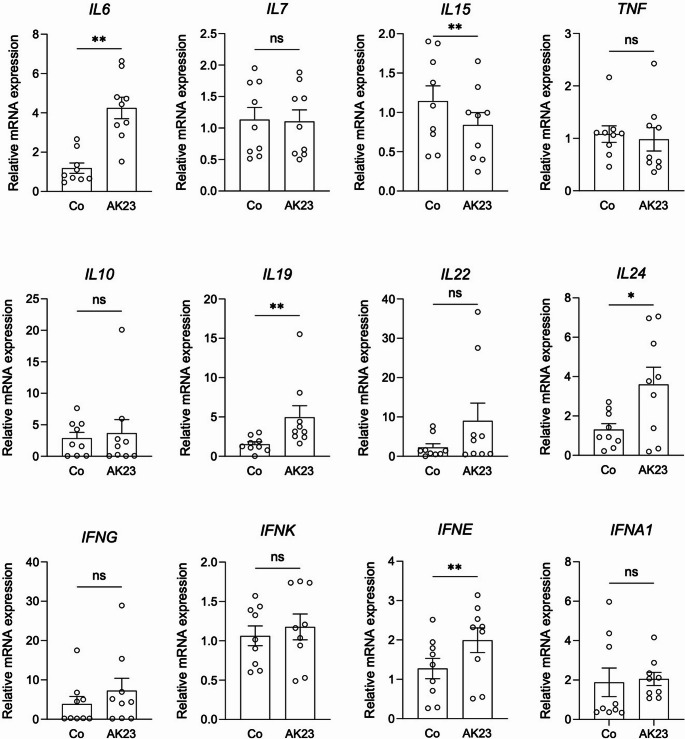
Anti-Dsg3 autoantibodies induce the expression of IL6, IL19, IL24 and IFNE in keratinocytes Relative cytokine expression in normal human epidermal keratinocyte (NHEK) monolayers before and after treatment with the anti-Dsg3 autoantibody AK23 for 4 h was determined by quantitative real-time PCR (n=9). Data were normalized to ACTB and POLR2A expression. Wilcoxon matched-pairs signed rank test was used for comparison. Values of *p* <0.05 were considered significant (*, *p* <0.05; ** *p* <0.01; ns, not significant)

First, we aimed to analyze, if the disruption of cell-cell contacts in keratinocytes induced by the binding of anti-Dsg3 antibody has any impact on cytokine expression. We treated NHEK monolayers with AK23 and analyzed the expression of cytokines expressed by keratinocyte (*n* = 9). The expression of *IL7*, *IL10*, *IL22* and *TNF* were not affected. However, the expression of *IL6*, *IL19* and *IL24* significantly increased in cells treated with AK23, while the expression of *IL15* significantly decreased (Fig. 1). When studying the expression of interferons (IFN) upon AK23 treatment, only the expression of *IFNE* showed a significant change (Fig. 1). Our results show that acantholysis induced by AK23 influences cytokine expression in keratinocytes (*IL6*, *IL15*, *IL19*, *IL24* and *IFNE*). These cytokines bind to type I (IL-6) or type II (IL-19, IL-24, IFN-ε) cytokine receptors and all use the JAK/STAT pathway for signal transduction.

### Binding of Dsg3 by Autoantibodies Induces STAT Activation in Keratinocytes

**Fig. 2:**
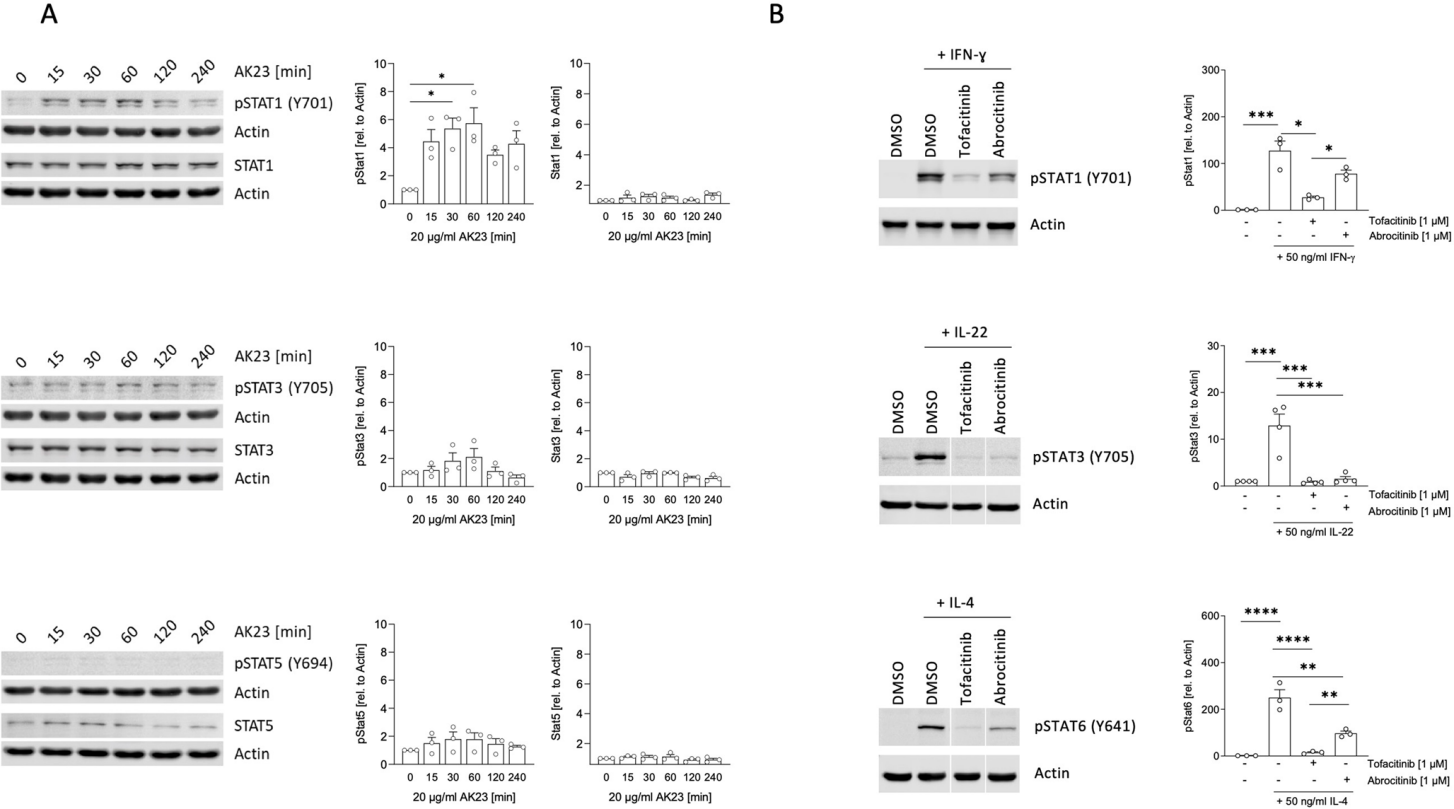
The JAK/STAT pathway in keratinocytes is activated upon autoantibody binding. **A**, Protein levels of non-activated and activated STAT1, STAT3 and STAT5 transcription factors after stimulation with the anti-Dsg3 autoantibody AK23. NHEK were incubated with 20 µg/ml AK23 for 0, 15, 30, 60, 120 and 240 min. Protein levels of total STAT1, STAT3 and STAT5 and phosphorylated STAT proteins were analyzed by immunoblotting (*n* = 3). Representative blots from one experiment and summarized data from three independent experiments are shown (mean ± SEM) Graphs show semi-quantitative analysis of signal intensity. Data were normalized to actin and control was set to 1 (0 min, buffer only). **B**, NHEK were activated for 30 min with interferon-γ, interleukin (IL-)22 or IL-4 (50ng/ml) to induce activation of STAT1 (*n* = 3), STAT3 (*n* = 4) or STAT6 (*n* = 3) respectively. DMSO (control) or 1 µM tofacitinib or abroticinib were pre-incubated for 1 h. STAT activation was analyzed by immunoblotting. Representative blots from one donor and summarized data from three independent experiments are shown. Graphs show semi-quantitative analysis of signal intensity. Dunn’s multiple comparison test after Friedman test (*, *p* < 0.05; **, *p* < 0.01; ***, *p* < 0.001; ****, *p* < 0.0001)

Since AK23 induced the expression of some JAK/STAT cytokines, we next analyzed STAT protein level and activation in keratinocytes 15, 30, 60, 120 and 240 min after AK23 binding (*n* = 3, Fig. 2A). We detected an early increase in pSTAT1 15 min upon AK23 binding and a peak after 60 min, followed by a subsequent decrease of the signal. Total STAT1 protein levels remained unchanged during the stimulation time. We also observed a weak activation of STAT3 after 60 min of AK23 treatment, without changes in total STAT3 protein levels. STAT4, STAT5 and STAT6 were not activated through AK23 treatment (*n* = 2, Fig. 2A and Supplementary Fig. S1). To demonstrate, that keratinocytes in principle respond to cytokine signals by STAT activation and that this signaling pathway can be reverted in keratinocytes by selective inhibitors, we stimulated NHEK cells with either IFN-ɣ (*n* = 3), IL-22 (*n* = 4) or IL-4 (*n* = 3) in the presence of either tofacitinib (Tofa) or abrocitinib (Abro). As expected, IFN-ɣ induced STAT1 activation, IL-22 activated STAT3 and IL-4 STAT6. The pan-JAK inhibitor Tofa prevented IFN-ϒ-induced STAT1 phosphorylation, IL-22-mediated STAT3 activation and IL-4-induced STAT6 activation. The JAK1 inhibitor Abro blocked IL-22-mediated STAT3 activation and reduced the phosphorylation of STAT1 and STAT6 after IFN- ɣ and IL-4 treatment, respectively (Fig. 2B). Our results show that autoantibody binding to Dsg3 results in STAT1 and to some extent to STAT3 activation. Moreover, the JAK/STAT pathway is active in keratinocytes and can be blocked efficiently by JAK inhibitors.

### JAK Inhibitors Prevent Autoantibody-mediated STAT Activation and Fragmentation in Keratinocytes

**Fig. 3:**
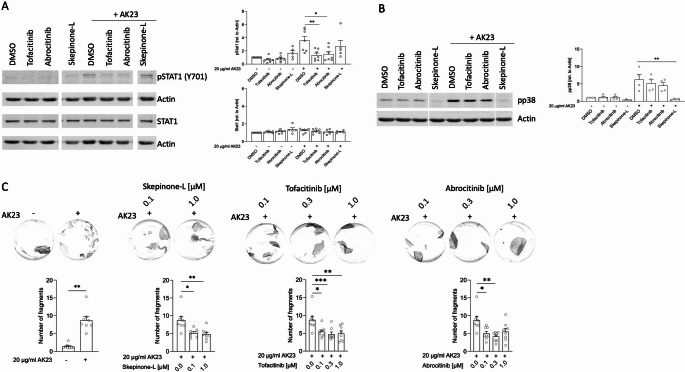
Janus kinase inhibitors prevent STAT1 activation and cell dissociation in keratinocytes treated with anti-Dsg3 autoantibody. **A**, NHEK were incubated with DMSO alone (control) or together with 1 µM tofacitinib, abrocitinib or skepinone-L for 1 h. Total and phosphorylated STAT1 protein levels were measured after presence or absence of 20 µg/ml of AK23 antibody for 4 h (*n* = 7, for skepinone-L *n* = 5). Representative blots from one donor are shown for pSTAT1, STAT1 and actin. Graphs show semi-quantitative analysis of signal intensity. Dunn’s multiple comparison test after Friedman test (*, *p* < 0.05; **, *p* < 0.01). **B**, NHEK were incubated with DMSO alone (control) or together with 1 µM tofacitinib, abrocitinib or skepinone-L and total and phosphorylated p38 protein levels were measured after presence or absence of 20 µg/ml AK23 antibody for 4 h (*n* = 4). A representative blot and summarized data from four independent experiments for pp38 and actin are shown. Dunn’s multiple comparison test after Friedman test (**, *p* < 0.01). **C**, Acantholysis was induced in NHEK monolayers by AK23 (20 µg/ml) for 4 h after 1 h pre-incubation with indicated concentrations of skepinone-L, tofacitinib or abrocitinib in DMSO or DMSO alone (control). Quantification was achieved by counting the number of fragments obtained after application of mechanical stress to the monolayers (*n* = 8). Representative images showing AK23 induced monolayer fragmentation of one experiment with each inhibitor as indicated as well as summarized data from 8 experiments are shown (mean ± SEM). Dots represent the mean fragmentation number of triplicates from each donor at the given conditions. Wilcoxon matched-pairs signed rank test for comparing control condition with and without AK23 treatment. Friedman test and Dunn’s multiple comparison test (* *p* < 0.05, ** *p* < 0.01, *** *p* < 0.001) for comparison of each condition with AK23 alone

To elucidate, whether the STAT activation in keratinocytes after autoantibody binding is a bystander effect or has functional impact, we treated NHEK cells with AK23 and followed the activation of STAT1 in the presence of pharmacological signaling inhibitors. The p38 mitogen-activated protein kinase (MAPK) inhibitor Skepinone-L was used as a control, since AK23 has been reported to initiate p38 MAPK activation in keratinocytes [17, 18]. Pretreatment of NHEK cells with Tofa (*n* = 7), Abro (*n* = 7) or Skepinone-L (*n* = 5) did not impact total STAT1 protein levels (Fig. 3A). Importantly, Tofa and Abro blocked STAT1 phosphorylation in keratinocytes treated with AK23 (Fig. 3A). Skepinone-L did not affect pSTAT1 but prevented p38 phosphorylation in AK23-treated NHEK. Tofa and Abro had no effect on p38 activation (*n* = 4, Fig. 3B). As demonstrated earlier, Skepinone-L treatment reduced keratinocyte monolayer fragmentation induced by AK23 in the well-established dispase dissociation assay (DDA) (*n* = 8, Fig. 3C) [6, 19]. Remarkably, Tofa and Abro also showed protective effects in keratinocytes in the DDA model and significantly diminished monolayer´s fragmentation through AK23 treatment (*n* = 8, Fig. 3C). Thus, NHEK fragmentation through AK23 treatment activates STAT1 and to some extent STAT3. JAK inhibitors like Tofa and Abro inhibit STAT1 activation and cell-cell fragmentation induced by Dsg3 autoantibodies.

### *STAT1* and *STAT3* Are Functional in keratinocytes, Do not Affect Dsg3 Protein Expression and Are Overrepresented in Pemphigus Skin

**Fig. 4:**
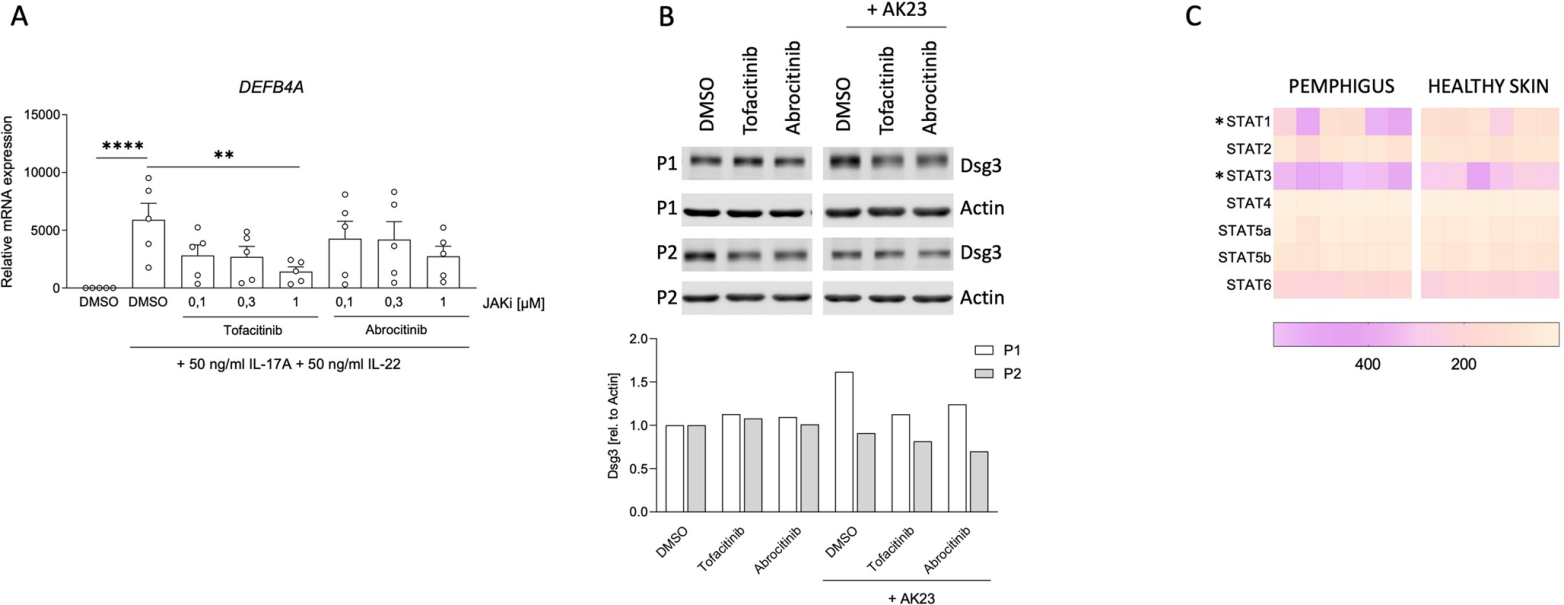
STAT1 and STAT3 are overexpressed in pemphigus skin and have regulatory function on gene expression but not on desmoglein protein levels.**A**, NHEK were activated for 30 min with 50ng/ml IL-22 and IL-17A after stimulation for 1 h with DMSO (control) or tofacitinib or abrocitinib at 0.1, 0.3 or 1µM concentration. Expression of *DFEB4A* was analyzed by quantitative real- time PCR (n=5). Dunn’s multiple comparison test after Friedman test (**, *p*<0.01, ****, *p*<0.0001). **B**, Western blot analysis of desmoglein 3 protein level in NHEK at steady state and after treatment for 4 h with 20 µg/ml AK23 alone or after pre-incubation for 1 h with either 1 µM tofacitinib or abrocitinib (n=2). Graph shows semi-quantitative analysis of signal intensity. **C**, Heatmap of STATs from whole transcriptome shotgun sequencing (RNA-seq) of pemphigus and healthy skin from a previously published data-set. The individual cell coloring depicts the counts per million (CPM) expression of the respective gene in the respective patients (pemphigus, n = 6; healthy controls n = 6). Mann-Whitney test (* p<0.05)

We further studied the direct functional impact of JAK inhibitors on keratinocytes and cell-cell fragmentation. When unstimulated, NHEK did not express *DEFB4A* (which encodes defensin β4A), while *s*timulation with IL-22 and IL-17 A resulted in a strong *DEFB4A* expression (*n* = 5, Fig. 4A). Since Tofa and Abro block IL-22-mediated STAT3 activation in keratinocytes (Fig. 2B), both JAK inhibitors impaired *DEFB4A* expression in NHEK cells stimulated with IL-22 and IL-17 A (Fig. 4A). Thus, cytokine-dependent JAK/STAT pathway is functional in keratinocytes. Dsg3 protein expression itself was not affected by JAK inhibition as demonstrated in Fig. 4B (*n* = 2). We next analyzed In-vivo-STAT expression levels in pemphigus skin by re-analyzing a previous RNA-sequencing (RNA-seq) data-set [13]. In pemphigus skin (*n* = 6), we observed a significantly stronger expression of *STAT1* and *STAT3* than in healthy skin (HC, *n* = 6). Other STAT members showed low and similar expression levels (Fig. 4C).

### JAK Inhibitors Prevent Human Anti-desmoglein Antibody Induced Keratinocyte Dissociation

**Fig. 5:**
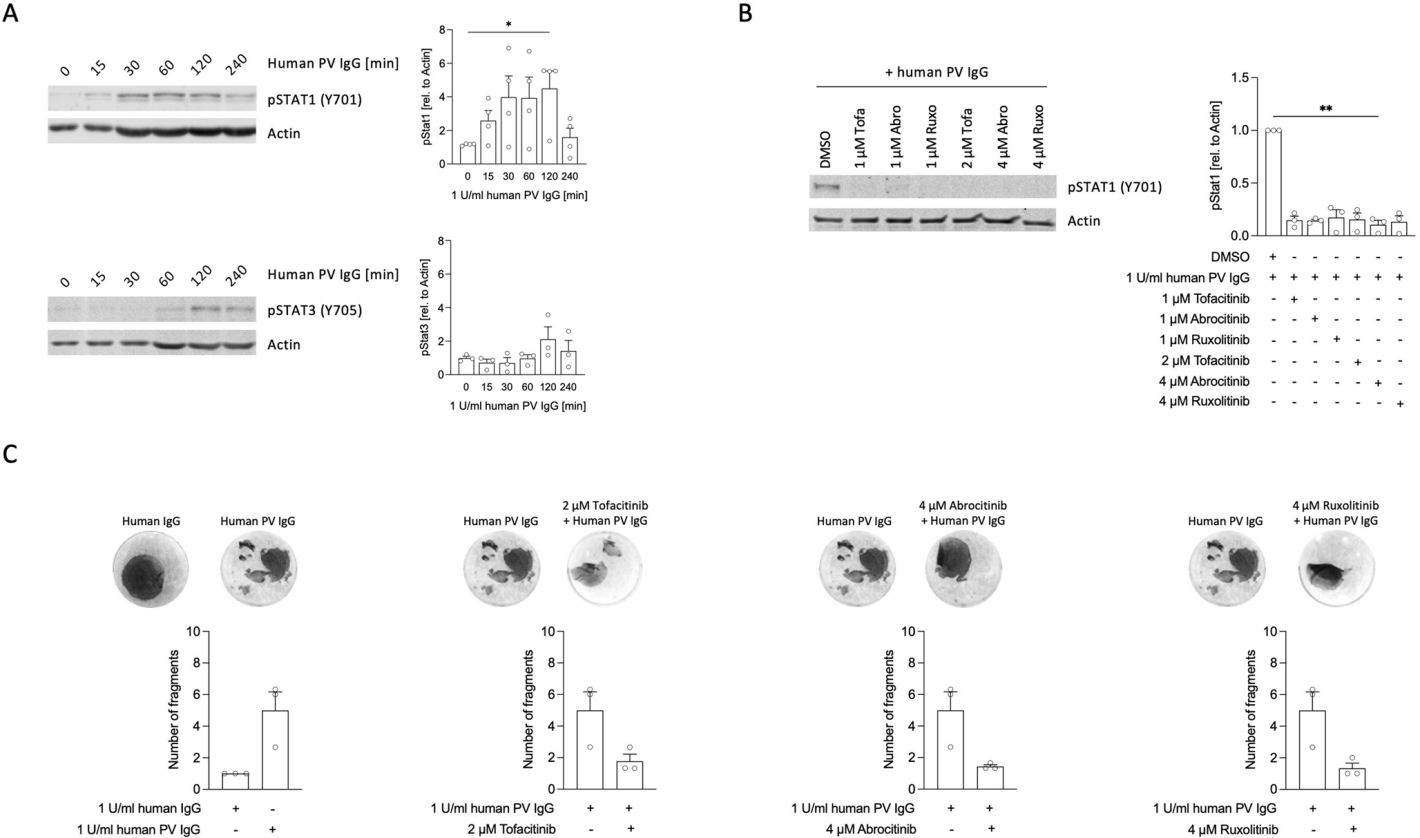
JAK inhibitors prevent STAT1 activation and cell dissociation in keratinocytes treated with human pemphigus sera. **A**, Protein levels of activated STAT1 and STAT3 after stimulation with human PV IgG. NHEK were incubated with 1 U/ml human pemphigus vulgaris IgG (PV IgG) for 0, 15, 30, 60, 120 and 240 min. Protein levels of phosphorylated STAT1 (*n* = 4) and STAT3 (*n* = 3) were analyzed by immunoblotting. Representative blots from one experiment and summarized data from three or four independent experiments are shown, respectively (mean ± SEM). Graphs show semi-quantitative analysis of signal intensity. Data were normalized to actin and control was set to 1 (0 min, buffer only). **B**, NHEK were treated for 120 min with 1 U/ml human PV IgG and preincubated with DMSO (control) or with tofacitinib, abrocitinib or ruxolitinib at either 1 µM, 2 µM (tofacitinib) or 4 µM (abrocitinib and ruxolitinib) concentration. STAT1 activation was analyzed by immunoblotting. Representative blots from one donor and summarized data from three independent experiments are shown. **C**, Acantholysis was induced in NHEK monolayers by human PV IgG (1 U/ml) or human serum IgG from healthy individuals for 24 h after 1 h pre-incubation with 2 µM of tofacitinib, 4 µM abrocitinib or 4 µM ruxolitinib in DMSO or DMSO alone (control). Quantification was achieved by counting the number of fragments obtained after application of mechanical stress to the monolayers (*n* = 3). Representative images showing monolayer fragmentation of one single experiment for each experimental setting as indicated. Bars show summarized data from 3 independent experiments (mean ± SEM). Dots represent mean fragmentation number of triplicates from each donor at the given conditions

To confirm the changes observed after treatment of NHEK with AK23, we analyzed STAT1 activation status of NHEK after 15, 30, 60, 120 and 240 min of incubation with purified IgG from patients with active PV (PV IgG). Similar to the observed changes with AK23 treatment, we observed an early increase in pSTAT1 expression in NHEK 15 min after PV IgG incubation with a significant peak at 120 min (*n* = 4, Fig. 5A). We also detected a weak pSTAT3 activation after 120 min (*n* = 3). Addition of the JAK inhibitors Tofa, Abro, and ruxolitinib (Ruxo) effectively suppressed PV IgG-mediated STAT1 activation (*n* = 3, Fig. 5B). Finally, we tested whether JAK inhibitors can prevent human PV IgG-induced NHEK fragmentation as observed with AK23. Experiments with PV IgG were performed to confirm the observed protective effect of JAK inhibitors from AK23-induced NHEK fragmentation. The limited number of experiments performed did not show statistical relevance, but all tested JAK Inhibitors (Tofa, Abro and Ruxo) showed marked inhibitory effects against human PV IgG-mediated fragmentation of NHEK monolayers (Fig. 5C).

### Epidermal Pemphigus Skin Shows High Levels of Activated STAT1 and STAT3

**Fig. 6:**
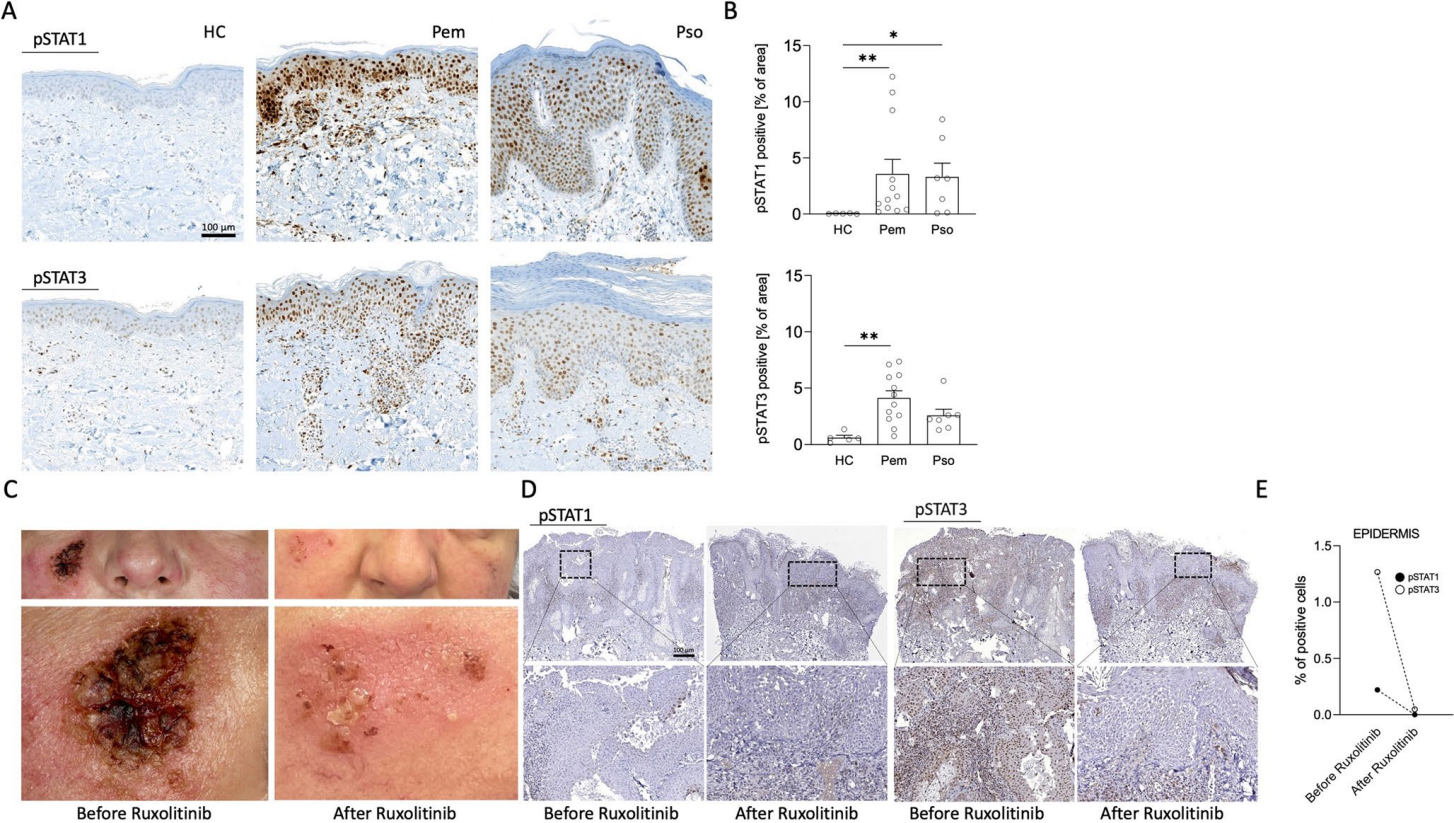
STAT1 and STAT3 are activated in pemphigus keratinocytes and their inhibition restores pemphigus epidermal structure. **A**, Activated STAT1 and STAT3 in the skin of pemphigus (Pem (PV and PF) *n* = 12), psoriasis (Pso, *n* = 7) and healthy individuals (*n* = 5) by immunostaining of formalin-fixed paraffin embedded tissue samples with antipSTAT1 (Y701) and anti-pSTAT3 (Y705) antibodies. Representative microscopic images for each group are shown (magnification 100x). **B**, Protein expression was determined by semi-quantitative analysis of the specific pSTAT signal in the epidermis. Single patients and mean standard error of mean (SEM) are plotted. Dunn’s multiple comparison test after Kruskal-Wallis test (*, *p* < 0.05; **, *p* < 0.01). **C**, Images of a chronic steroid-refractory lesion (left) treated for 12 weeks with the topical Janus kinase inhibitor ruxolitinib (right). **D**, Immunohistological analysis of pSTAT1 and pSTAT3 expression in biopsies from the lesion as described in C before and after treatment with ruxolitinib (magnification 100x for upper row, 400x for lower row). **E** Graph shows quantified epidermal protein expression of STAT1 and STAT3. HC healthy control, PEM pemphigus, PSO psoriasis

We next analyzed the activation status of STAT1 and STAT3 in the lesional epidermis of pemphigus skin (Pem, *n* = 12) compared to the Th17-disease psoriasis (Pso, *n* = 7) and HC (HC, *n* = 5). Phosphorylated STAT1 and STAT3 were minimally present in HC skin keratinocytes. As reported previously, pSTAT1 and pSTAT3 were observed in psoriatic keratinocytes [20]. Interestingly, we also observed a strong presence of pSTAT1 and pSTAT3 in the epidermis of pemphigus skin samples (Fig. 6A and B). The expression of pSTAT1 and pSTAT3 in epidermal cells was comparable between the two main pemphigus subtypes pemphigus vulgaris (PV) and pemphigus foliaceus (PF) (Supplementary Fig. S2). Our immunohistological findings in vivo confirm our in vitro results on STAT1 and STAT3 activation in keratinocytes in the presence of autoantibody binding to Dsg3.

### Topical Application of the JAK Inhibitor Ruxolitinib Restores cell-cell Integrity in Pemphigus Skin

Finally, we decided to treat one pemphigus patient with chronic steroid-refractory skin lesions (patients´ characteristics are resumed in supplementary table S3) with a JAK inhibitor. To primarily target the epidermal compartment, we decided to apply a topical JAK inhibitor. The JAK1/JAK2 inhibitor Ruxo is approved for the treatment of vitiligo and atopic dermatitis in some countries [21]. Ruxo creme (Opzelura™) was applied twice daily as monotherapy for 12 weeks in a compassionate-use setting. Informed consent was obtained from the patient prior to inclusion in this study. Topical JAK inhibitor treatment strongly led to complete re-epithelization of the before steroid-unresponsive chronic lesion (Fig. 6C). Histological examination before and after treatment revealed that the clinical improvement and re-epithelization with compact cell-cell integrity were accompanied by an epidermal decrease in pSTAT1 and pSTAT3 (Fig. 6D and E). Taken together, our data show that autoantibody binding to Dsg3 activates the JAK/STAT pathway and contributes to epidermal instability. JAK inhibitors both in vitro and in vivo block JAK/STAT activation in keratinocytes and sustain epidermal stability.

## Discussion

In this exploratory study, we demonstrate that autoantibody binding to Dsg3 activates keratinocytes to express cytokines that use the JAK/STAT pathway for signal transduction. Importantly, anti-Dsg3 autoantibody binding results in the activation of STAT1 and to some extent STAT3. The slightly different kinetics of STAT1 and STAT3 phosphorylation observed after use of AK23 or PV IgG are most likely due to the fact that AK23 is a monoclonal Dsg3 autoantibody derived from a hybridoma cell line, whereas PV IgG contains polyclonal IgG with multiple epitope specificities. In pemphigus lesional skin, where autoantibodies to Dsg3 are typically attached to keratinocytes, we detected activated STAT1 and STAT3 in epidermal keratinocytes. By using JAK inhibitors we could prevent cytokine-induced STAT activation and STAT-dependent gene expression in keratinocytes. Most importantly, we could inhibit autoantibody-induced STAT activation and disruption of cell-cell interaction in keratinocyte monolayers by using JAK inhibitors. These protective effects by JAK inhibitors were observed in keratinocytes treated with either AK23 or PV IgG. Application of a topical JAK inhibitor clinically improved a refractory pemphigus lesion, reduced STAT1 and STAT3 activation in the epidermis and restored epidermal intercellular adhesion.

Our RNA studies show that autoantibody binding to Dsg3 induces a significant increase in *IL6*, *IL19*, *IL24* and *IFNE* expression (Fig. 1). In a previous study, we could demonstrate that these cytokines are typically found in the skin of patients with pemphigus [13]. These cytokine data are in concordance with our Western blot data showing STAT activation after Dsg3 autoantibody binding (Figs. 2A and 5 A). The IL-6 receptor signals through JAK1/JAK2, activates STAT3 and to a lesser extent STAT1 [22]. IL-6 produced from keratinocytes promotes inflammation and migration of inflammatory cells [23]. IL-19 and IL-24, signal through the IL-20 cytokine receptor, which associates with JAK1/TYK2 and downstream activates STAT3 and to a lesser extent STAT1 for signaling [24]. IL-19 and IL-24 are typically produced from keratinocytes, act in an autocrine manner and promote pro-inflammatory responses [11, 25]. IFN-ɛ, which belongs to the family of type I IFNs binds to the IFNAR receptors subunit I and II, which signal through JAK1/TYK2. The dominant downstream transcription factor of IFNAR is STAT1 [26]. IFN-ɛ has been suggested to contribute to anti-viral, anti-proliferative and immunoregulatory responses [27]. In humans, the induction of these aforementioned cytokines following anti-Dsg3 autoantibody binding in the epidermis might help to promote an inflammatory milieu and lymphocyte recruitment [10]. By using JAK inhibitors we could silence STAT1 activation and subsequent responses (Fig. 2B and 4A). The pan-JAK inhibitor Tofa seems to be more potent in blocking AK23-induced responses in keratinocytes than Abro and Ruxo, which are more selective JAK inhibitors [28]. 

Our in vitro data with NHEK and the DDA showed a more prominent STAT1 activation than STAT3 activation (Figs. 2A and 5A). In agreement with our findings, recently published studies support our findings on a functional role of the JAK/STAT pathway in pemphigus skin pathology. Chen and colleagues reported increased expression of phosphorylated JAK1 (pJAK1) and pJAK2 in keratinocytes of lesional pemphigus skin and in immortalized human keratinocyte cell lines (HaCaT) after incubation with PV IgG sera. Ruxo reduced apoptosis and endoplasmic reticulum (ER) stress in HaCaT cells. A second study used a proteomics approach and identified increases in STAT1, pSTAT1 and pJAK1 in HaCaT cells treated with PV IgG sera [29, 30]. However, our RNA-seq and IHC data from pemphigus skin in vivo also revealed a strong STAT3 activation (Figs. 4C and 6A). This discrepancy between in vitro and in vivo findings is best explained by the fact that in vivo, immune cells also infiltrate the skin and release cytokines, that activate both STAT3 and STAT1. Consistent with the concept of activated JAK/STAT pathway in pemphigus skin, previous analyses also described an overexpression of STAT4 and STAT6 in pemphigus epidermal cells [31]. Our preliminary analysis on two patients also revealed that JAK inhibitors do not affect Dsg3 expression in NHEK (Fig. 4b). This result will need further exploration on larger cohorts to better address potential heterogeneity among patient-derived samples.

Our IHC and transcriptome data show that pemphigus skin expresses significantly higher levels of *STAT1*/pSTAT1 and *STAT3*/pSTAT3 than healthy skin (Figs. 4C and 6A). Expression levels of pSTAT1 and pSTAT3 were comparable to those seen in psoriasis skin. In patients with psoriatic arthritis, Tofa is already approved by the American and European medicines agencies (FDA, EMA) and improves psoriatic skin as demonstrated in phase 3 trials [32, 33]. In pemphigus, so far therapeutical trials with JAK inhibitors have not been published, although Tofa has been reported to suppress autoantibody development in preclinical studies and Ruxo demonstrated protective effects on preserving HaCaT cells from intercellular junction loss when treated with anti-Dsg antibodies. Finally, Ruxo 5 mg daily has been described to be effective in one patient with paraneoplastic pemphigus [30, 34, 35]. Pemphigus pathogenesis is complex and apparently driven by different types of cytokines and immune cells (T and B cells). Some of the cytokines involved in pemphigus pathogenesis signal through the JAK-STAT pathway others don’t (i.e. IL-1, IL-17) [13, 36, 37]. Monoclonal antibodies targeting specific cytokines have not been established so far for the treatment of pemphigus [36]. Our findings from treating a skin lesion of a patient with chronic and steroid-unresponsive pemphigus demonstrate that topical application of a JAK inhibitor has a beneficial impact on skin lesions (Fig. 6C and D). In a preclinical pharmacokinetic study topical application of Ruxo showed to have a better cutaneous distribution than systemic administration [38]. This is of interest, given the limited efficacy of topical steroids in pemphigus [39]. Topical JAK inhibitors that interfere with anti-Dsg3 autoantibody-mediated STAT activation may serve as a new therapeutic alternative in treatment-resistant pemphigus lesions. Yet, studies on murine pemphigus models and larger patient cohorts are needed to validate and extend our findings and to determine a potential therapeutic benefit of JAK inhibition in pemphigus.

In summary, here we show that antibody binding to Dsg3 disrupts epidermal integrity and activates immune pathways in keratinocytes with an increase in phosphorylated STAT1 and STAT3 and an increase in expression levels of *IL6*, *IL19*, *IL24* and *IFNE*, which signal through STAT1 and STAT3. Application of JAK inhibitors in both in vitro and in vivo have a protective role, silence STAT activation, gene expression in keratinocytes and prevent epidermal acantholysis.

## Supplementary Information

Below is the link to the electronic supplementary material.

ESM 1 Supplementary File 1 (DOCX 18.4 KB)

ESM 2 Supplementary File 2 (DOCX 16.0 KB)

ESM 3 Supplementary File 3 (JPG 171 KB)

ESM 4 Supplementary File 4 (JPG 382 KB)

## Data Availability

Dataset from Holstein et al. [13] with GEO accession number GSE299468 was analysed during the current study (Fig. 4C).
