# Supplementary material for: Inhibition of the JAK/STAT Signaling Pathway Suggests a Protective Effect against Acantholysis in Pemphigus
Source: Inflammation. 2026 Jan 5;49(1):89. doi: 10.1007/s10753-025-02417-y (PMC12956940; doi:10.1007/s10753-025-02417-y)
Supplement: Supplementary file 1 — (DOCX 18.4 KB) [file 10753_2025_2417_MOESM1_ESM.docx]

Table S1: Pemphigus patients for immunohistochemical analysis

| **Patient** | **Sex** | **Age** | **Pemphigus type** | **Antibody level**  **Dsg1/Dsg3**  **[RU/ml]** | **Disease status** |
| --- | --- | --- | --- | --- | --- |
| 1^a^ | F | 43 | PF | >200/4 | Acute |
| 2 | M | 72 | PV | >200/135 | Acute |
| 3 | M | 75 | PF | >200/5 | Acute |
| 4^a^ | M | 50 | PV | >200/180 | Chronic |
| 5 | M | 87 | PF | >200/<2 | Chronic |
| 6 | M | 66 | PF | >200/4 | Chronic |
| 7 | F | 60 | PV | 60/>200 | Chronic |
| 8 | F | 51 | PV | 116/>200 | Chronic |
| 9 | F | 66 | PF | >200/<2 | Chronic |
| 10 | M | 50 | PV | >200/>200 | Chronic |
| 11 | M | 57 | PF | 81/19 | Chronic |
| 12 | M | 36 | PV | 189/>200 | Chronic |

^a^ Representative images for figure 6A

**Table S2:** Demographics of pemphigus sera used for in vitro experiments

| **Patient** | **Sex** | **Age** | **Pemphigus type** | **Antibody level**  **Dsg1/Dsg3**  **[RU/ml]** | **Disease status** |
| --- | --- | --- | --- | --- | --- |
| 1 | M | 79 | PV | 91/152 | Chronic |
| 2 | F | 57 | PV | 44/>200 | Chronic |
| 3 | M | 44 | PV | 118/>200 | Chronic |
| 4 | M | 36 | PV | >200/>200 | Chronic |

**Table S3:** Demographics of a pemphigus patient treated with a topical JAK 1/2 inhibitor

| **Sex** | **Age** | **Pemphigus Type** | **Antibody level**  **Dsg1/Dsg3 (RU/ml)** | **Disease status** | **Therapy** | **ABSIS before**  **Treatment** | **ABSIS after**  **Treatment** |
| --- | --- | --- | --- | --- | --- | --- | --- |
| F | 59 | Pemphigus vulgaris | 3/148 | Chronic | Dapson 150mg/daily  Prednisone 5mg/daily | 1,5 | 0,5 |

ABSIS, Autoimmune Bullous Skin Disorder Intensity Score; RU/ml relative units per milliliter; Dsg desmoglein
