# Supplementary material for: Inhibition of the JAK/STAT Signaling Pathway Suggests a Protective Effect against Acantholysis in Pemphigus
Source: Inflammation. 2026 Jan 5;49(1):89. doi: 10.1007/s10753-025-02417-y (PMC12956940; doi:10.1007/s10753-025-02417-y)
Supplement: Supplementary file 2 — (DOCX 16.0 KB) [file 10753_2025_2417_MOESM2_ESM.docx]

**Appendix S1**

List of Primers

*ACTB*: for AgCCTCgCCTTTgCCgA, rev CTggTgCCTggggCg, probe CCgCCgCCCgTCCACACCCgCC

*POLR2A*: for gTggAgATCTTCACggTgCT, rev gTgCggCTgCTTCCATAA, probe TACCACgTCATCTCCTTTgATggCTCCTAT

*IL6*: for CCAgAgCTgTgCAgATgAgTACA, rev CCTgCAgCTTCgTCAgCA, probe CATTTgTggTTgggTCAggggTggT

*IL7*: for ACTATgggCggTgAgAgCT, rev TggCAACAgAACAAggATCA, probe CCTCCCgCAgACCATgTTCCA

*IL15*: for AAATTgAAgATCTTATTCAATCTATgCA, rev CACTTCATTgCTgTTACTTTgCA, probe ACggAAAgTgATgTTCACCCCAgT

*TNF*: for CTTCTCCTTCCTgATCgTggC, rev gggTTTgCTACAACATgggC, probe CgCCACCACgCTCTTCTgCCT

*IL10*: for gCTACggCgCTgTCATCgA, rev AgATgCCTTTCTCTTggAgCTTA, probe ACCTgCTCCACggCCTTgCT

*IL19*: for CTgCggCAATgTCAggAAC, rev CgTggACCTCCAgCTgATCATA, probe ATgACTCTggTggCATTggTggC

*IL22*: for TgATgACCTgCATATCCAgAggAAT, rev ATCCAgTTCTCCAATTgCTTTgATC, probe TgCAAAAgCTgAAggACACAgTgAAAAA

*IL24*: for AgCATTCAAACAgTTggACgTA, rev TCTAgACATTCAgAgCTTgTAgAATTT, probe CAAgggCTTTggTCAgAgCTgC

*IFNG*: for gCATCCAAAAgAgTgTggAg, rev ggACATTCAAgTCAgTTACCgA, probe ATCAAggAAgACATgAATgTCAAgTTTTTCAA

*IFNK*: for CTCTATTCAggAggAAATAAgAATCATCTA, rev CAggTACATTTCAgATATATTTCACCC, probe CAAgAATTAACAgAgATTgTggCTACgCAA

*IFNE*: for ggATAAgTAgCATATTTgACCTTCACC, rev CTTTCTTgATTCACTTgTCTTTgC, probe CTgCTggCCTCTACCACTATCTTCTCTC

*IFNA1*: for gAgTCACCCATCTCAgCAAgC, rev gAgCATCAAggTCCTCCTgTTA, probe AgCTgCAAgTCAAgCTgCTCTCTgg

*DEFB4A*: for CTgATgCCTCTTCCAggTgTTT, rev CTggATgACATATggCTCCACTC, probe TggTATAggCgATCCTgTTACCTgCC

**Reagents**

Tofacitinib citrate and Ruxolitinib phosphate were obtained from Selleck Chemicals (Houston, USA). The JAK1 selective inhibitor Abrocitinib was obtained from Sigma-Aldrich (St. Louis, USA). Skepinone-L was kindly provided by M. Forster from the Pharmaceutical Chemistry Department of the Tübingen University. IgG containing supernatants from the AK23 hybridoma cell line were kindly provided by R. Tikkanen, Justus Liebig University Giessen, Germany. Sera from PV patients and healthy individuals were collected at the Department of Dermatology, Venereology and Allergology, Charité - Universitätsmedizin Berlin. Purification of AK23, human IgG and PV IgG was performed according to Beckert et al. (1) The activity of purified PV IgG was measured by dispase dissociation assay and the amount of PV IgG generating a similar fragmentation as 20 µg/ml AK23 was defined as one Unit.

qPCR parameters

| Step | | Temperature | Time | Cycles | Analysis Mode |
| --- | --- | --- | --- | --- | --- |
| Enzyme activation | | 95 °C | 10 min | 1 | None |
| Amplification | Denaturation | 95 °C | 10 s | 50 | Quantification |
|  | Annealing and elongation | 60 °C | 20 s |  |  |
| Cool | | 40 °C |  | 1 | None |

Supplementary references:

Beckert B, Panico F, Pollmann R, Eming R, Banning A, Tikkanen R. Immortalized Human hTert/KER-CT Keratinocytes a Model System for Research on Desmosomal Adhesion and Pathogenesis of Pemphigus Vulgaris. Int J Mol Sci. 2019 Jun 26;20(13):3113. doi: 10.3390/ijms20133113.
