# Supplementary figures and images for: Inhibition of the JAK/STAT Signaling Pathway Suggests a Protective Effect against Acantholysis in Pemphigus
Source: Inflammation. 2026 Jan 5;49(1):89. doi: 10.1007/s10753-025-02417-y (PMC12956940; doi:10.1007/s10753-025-02417-y)

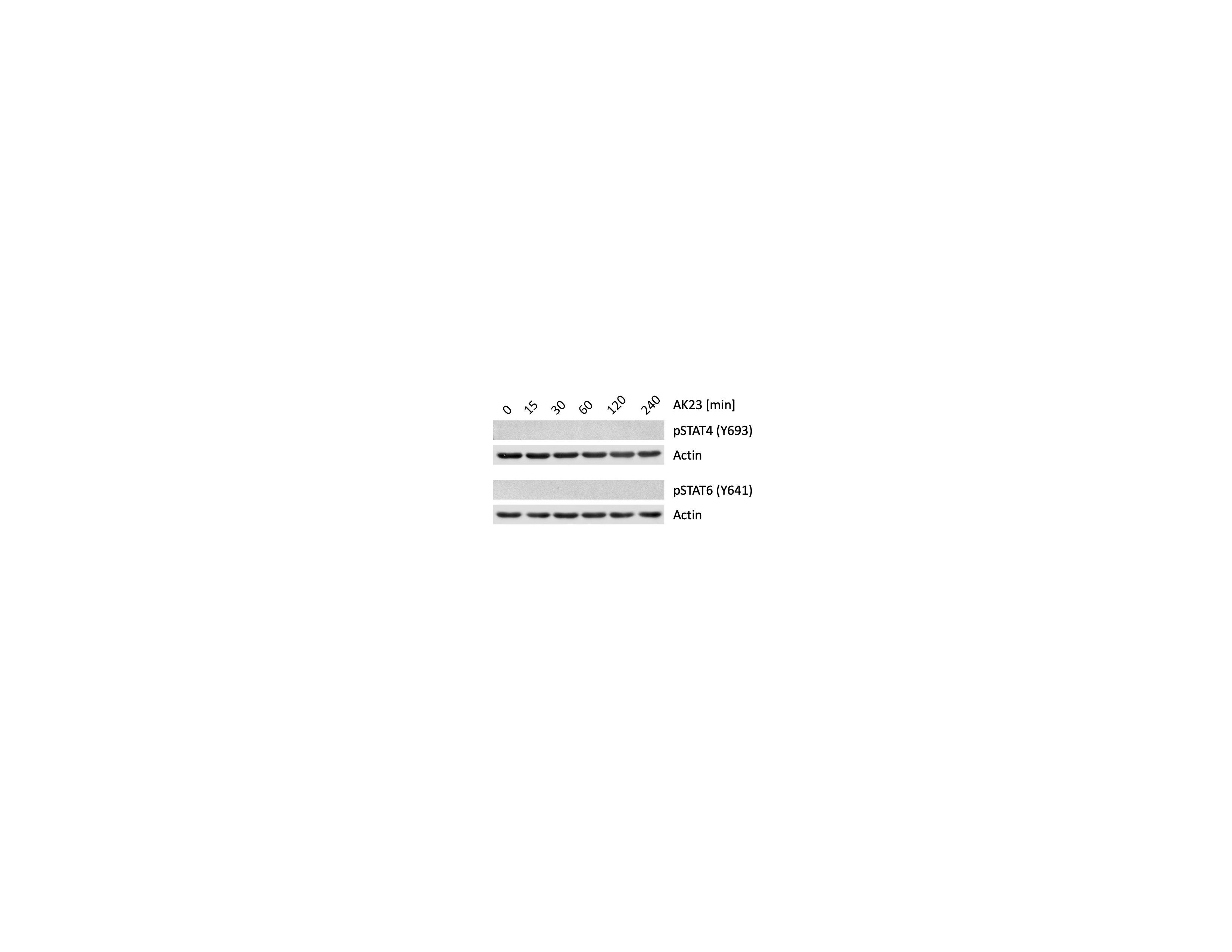

Supplement: Supplementary file 3 — (JPG 171 KB) [file 10753_2025_2417_MOESM3_ESM.jpg]

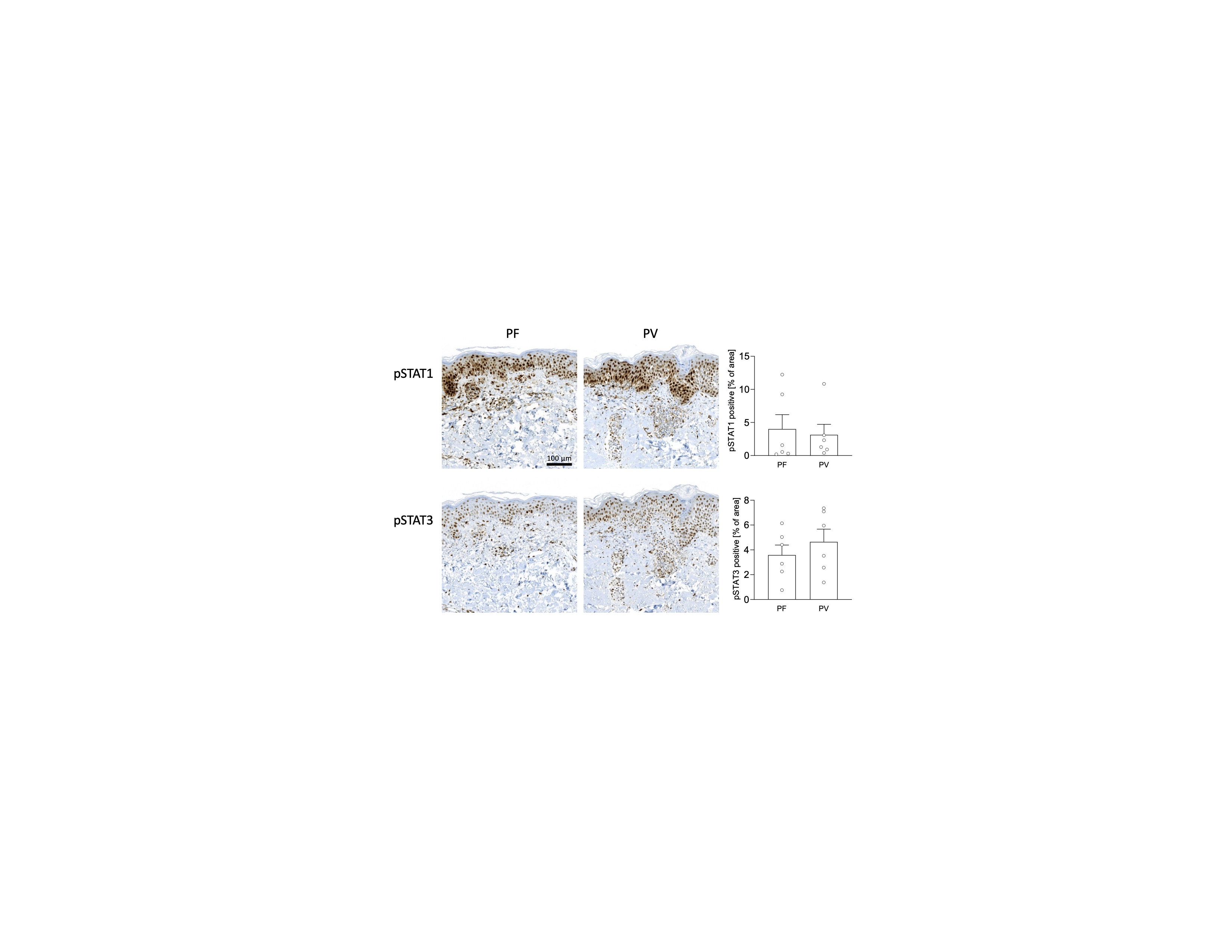

Supplement: Supplementary file 4 — (JPG 382 KB) [file 10753_2025_2417_MOESM4_ESM.jpg]
